# Endoplasmic reticulum microenvironment and conserved histidines govern ELOVL4 fatty acid elongase activity

**DOI:** 10.1194/jlr.M045443

**Published:** 2014-04

**Authors:** Sreemathi Logan, Martin-Paul Agbaga, Michael D. Chan, Richard S. Brush, Robert E. Anderson

**Affiliations:** * Departments of Cell Biology University of Oklahoma Health Sciences Center, Oklahoma City, OK 73104; § Ophthalmology, University of Oklahoma Health Sciences Center, Oklahoma City, OK 73104; † Dean McGee Eye Institute, Oklahoma City, OK 73104

**Keywords:** mutant, lipid metabolism, retinal degeneration, autosomal dominant, elongation of very long chain fatty acids-like 4

## Abstract

Autosomal dominant Stargardt-like macular dystrophy (STGD3) in humans results from mutations in elongation of very long chain FAs-like 4 (*ELOVL4*), which leads to vision loss in young adults. ELOVL4 is an integral endoplasmic reticulum (ER) protein that mediates the elongation of very long chain (VLC) FAs. Mutations in *ELOVL4* lead to truncation and mislocalization of the translated protein from the ER, the site of FA elongation. Little is known about the enzymatic elongation of VLC-FAs by ELOVL4. We over-expressed full-length mouse ELOVL4, an N-glycosylation-deficient mutant, an ER-retention mutant, and mutants of active site histidines to parse their individual roles in VLC-FA elongation. ELOVL4 elongated appropriate precursors to the corresponding VLC-FA species ≥28 carbons. Active site histidine mutants of ELOVL4 did not elongate appropriate precursors, establishing ELOVL4 as the elongase. Displacing ELOVL4 from the ER was sufficient to cause loss of condensation activity, while absence of N-glycosylation was irrelevant for enzyme function. This study shows that ELOVL4 enzymatic activity is governed by individual histidines in its active site and the ER microenvironment, both of which are essential for elongation of VLC-FAs.

The etiology of autosomal dominant Stargardt-like macular dystrophy (STGD3) in humans was described by three independent research groups as resulting from mutations in the elongation of very long chain fatty acids-like 4 (*ELOVL4*) gene (1–3). Dominant mutations in patients with STGD3 lead to early onset loss of central vision with progressive degeneration of the macula and subsequently the peripheral retina. Patients with homozygous mutations in *ELOVL4* exhibit severe skin and brain dysfunction (4). Truncating mutations in the last exon of *ELOVL4* cause a frame shift, leading to premature termination of the encoded protein and loss of its C-terminal endoplasmic reticulum (ER) retention signal. The truncated gene product is subsequently mislocalized to other cellular compartments, resulting in protein aggregation (5–7). Bearing sequence homology with the yeast *Elo* family of FA elongases, mammalian ELOVL4 has been shown to be involved in the biosynthesis of very long chain (VLC) FAs greater than 26 carbons in length (8, 9).

Elongation of FAs occurs through the cooperation of several ER-resident enzymes. The initial rate-limiting condensation reaction determining the chain length of the FA is catalyzed by an elongase, yielding a 3-keto-acyl-CoA intermediate (10–12). This intermediate product subsequently undergoes reduction catalyzed by 3-keto-acyl-CoA reductase (KAR), followed by dehydration by any of four different 3-hydroxylacyl-CoA dehydratases (HACD1, HACD2, HACD3, and HACD4) (13). The final reduction step yielding the elongated FA product is catalyzed by *trans*-2,3-enoyl-CoA reductase (TER) (14). ELOVL4 has been shown to associate with KAR and TER (15) and HACD1 (16) when coexpressed in culture. HACD1 has also been shown to coimmunoprecipitate with KAR and TER, thereby supporting the existence of an elongase complex (16).

VLC-FAs greater than 26 carbons (>C26) are unique in that they are found only in tissues expressing ELOVL4 such as retina, brain, testes, and skin (17), with the highest expression in the retina. VLC-PUFAs are enriched in phosphatidylcholine of retina (18) and brain (19) lipids, and in the SM and ceramide in testis and sperm (20). The VLC saturated FAs, on the other hand, are found in the SM and ceramide fractions in skin (21–24), where they function to prevent dehydration. Interestingly, these FAs are not normally detectable in the blood and liver, suggesting that they are produced locally from long chain FA precursors. However, they have been found in the blood of patients with adrenoleukodystrophy (25) and Zellweger syndrome (19, 26–28), both of which are peroxisomal disorders. In the retina, VLC-PUFAs have been shown to be tightly associated with rhodopsin, and were suggested to possibly aid in phototransduction (29). Due to similarities in the pathologies of STGD3 and autosomal recessive Stargardt's disease (STGD1), it was proposed that VLC-PUFAs in the retina may influence the flippase activity by the ABCA4 protein, the STGD1 disease gene product (30). Thus, it is crucial to have a better understanding of the activity of ELOVL4 and the VLC products it generates, which are important for skin, brain, and retinal function.

We have confirmed that ELOVL4 mediates the rate-limiting condensation reaction in VLC-FA elongation (9), specifically 26:0 to 28:0 (8). However, it is not known whether ELOVL4 can mediate successive elongation of other VLC-FAs, such as those generated in culture (8), as well as those present in the retina (18, 31). Furthermore, little is known about the individual protein motifs in ELOVL4 and their influence on VLC-FA elongation. Characteristic of other elongase and desaturase enzymes, ELOVL4 has three distinctive motifs along the length of the protein that may participate in VLC-FA biosynthesis: *1*) ELOVL4 has an N-glycosylation consensus site at the N terminus (5), possibly aiding proper folding and structural stability of the protein; *2*) ELOVL4 contains an active site comprising a histidine-rich motif (HXXHH) proposed to chelate iron, which acts as an electron transfer moiety during O_2_-dependent redox reactions (32); and *3*) a dilysine ER retention motif at the C terminus that localizes ELOVL4 to the ER, where KAR, TER, and HACD1–4 are also located. Lysine residues juxtaposed to the C terminus of transmembrane proteins provide a recognition signal for the ER retention machinery to allow retrieval and retention of ER-resident proteins (33). The dependence of ELOVL4-mediated elongation of VLC-FAs on the histidine residues, N-glycosylation status, or retention to the ER is unclear.

Hence, in this study we sought to characterize the enzymatic activity of the ELOVL4 protein and the contribution of each of the protein motifs toward the synthesis of VLC-FAs. We hypothesized that ELOVL4 is indeed the elongase that mediates successive elongation steps generating FA chain lengths ≥28 carbons, and that these individual motifs are critical for enzyme function. Using a gain-of-function approach, we show conclusively that ELOVL4 is the elongase responsible for elongation of FAs to C28, C30, C32, and C36 FAs. We also show that this activity is dependent on the coordination of the histidine residues in the active site and requires localization to the ER, while N-glycosylation is dispensable for enzyme function.

## EXPERIMENTAL PROCEDURES

### Cell culture

ARPE19 cells were cultured in 10 cm^2^ plates with DMEM-nutrient mixture F-12 (DMEM-F-12; Invitrogen, Grand Island, NY) supplemented with 10% heat-inactivated FBS (v/v; Aleken Biologicals, Nash, TX) and antibiotics [100 units/ml each of penicillin and streptomycin (Invitrogen)]. HEK293T and HeLa cells were grown in DMEM medium supplemented with 10% FBS and antibiotics.

### *Elovl4* plasmids, mutagenesis, and adenoviral constructs

Mouse *Elovl4* (WT and 5 bp deletion) were PCR amplified and cloned in frame with a triple hemagglutinin (HA) tag in *pKH^3^ BSENX* vector (kindly provided by Dr. Scott M. Plafker). Active site mutants of WT ELOVL4 were constructed by mutating single histidines to glutamine or by mutating all three histidines to glutamines. N-glycosylation mutant was constructed by mutating the NDTV consensus site to NDAV and the lysine mutant was constructed by mutating the two lysines in the ER retention signal to arginines. All mutagenesis was performed using QuickChange II site directed mutagenesis kits (Agilent Technologies Inc., Santa Clara, CA). Adenovirus particles (Ad5) were generated as previously described (9) using the RAPAd CMV adenoviral expression system (Cell Biolabs, Inc., San Diego, CA).

### Antibodies

We used the C-terminal rabbit polyclonal ELOVL4 antibody (C-ELOVL4) at 1:1,000 dilution to detect untagged ELOVL4 as reported in Agbaga et al. (8). In this study, we used mouse anti-HA (Cell Signaling Technology, Inc., Danvers, MA), rabbit anti-HA (Clonetech, Mountain View, CA), mouse anti-β actin (Abcam, Cambridge, MA), mouse anti-calnexin (Abcam), and rabbit anti-Green Fluorescent Protein (GFP) (Sigma-Aldrich, St. Louis, MO) antibodies.

### Immunoblotting

Cell pellets were lysed in lysis buffer containing 20 mM Tris-HCl (pH 7.4), 100 mM NaCl, 1 mM EDTA, complete protease inhibitors (EDTA free) (Roche, Mannheim, Germany), 1 mM PMSF, and 1% Triton X-100, and processed as previously described (9). Equal amounts of protein were separated on 12% polyacrylamide gels by SDS-PAGE and transferred to nitrocellulose membranes. Membranes were blocked with 5% nonfat dry milk and incubated with primary antibody overnight, followed by horseradish peroxidase-conjugated secondary goat anti-mouse or donkey anti-rabbit IgG for 1 h at room temperature. Immunoreactivity was detected by chemiluminescence using Super-Signal West Femto maximum sensitivity substrate (Pierce, Rockford, IL). Membranes were reprobed as necessary for the various markers.

### Immunocytochemistry

HeLa cells were grown on Labtek chamber slides and transiently transfected with ELOVL4 constructs. After 48 h, slides were rinsed and fixed as per Logan et al. (9). The slides were blocked with 5% nonfat dry milk and incubated with primary rabbit anti-HA antibody (Clonetech) and mouse anti-calnexin antibody (Abcam) overnight at 4°C. The following day, cells were washed and incubated with secondary anti-rabbit antibody conjugated with Alexa Fluor® 488 dye (Invitrogen) and anti-mouse antibody conjugated with Alexa Fluor® 568 dye (Invitrogen). The slides were then washed and coverslipped with Vectashield with DAPI mounting medium (Vector Labs) and imaged by confocal microscopy (Olympus FluoView 500, Olympus, Melville, NY).

### FA treatment

ARPE19 cells and HEK293T cells were transduced overnight with mouse ELOVL4 adenovirus and treated with FA precursors as described in Logan et al. (9). Sodium salts of the FAs were conjugated with BSA fraction V (Sigma) in a ratio 2:1 (w/w) BSA:FA for 20:5n3 (EPA) and 1:1 (w/w) BSA:FA for 26:0, 28:0, 30:0, and 34:5n3. Cells were treated with 30 μg/ml of the FA in media for a period of 48 h (HEK293T) or 72 h (ARPE19) unless otherwise stated. Following treatment, cells were harvested, washed once in 0.1 M PBS containing 50 μM of BSA fraction V (Sigma) to sequester excess free FAs, and washed with PBS only. The cell pellets were stored at −80°C until further processed for lipid analysis.

### Lipid extraction and separation

Lipids were extracted from cells and other tissues according to the procedure described by Bligh and Dyer (34). FA methyl esters (FAMEs) were generated by acid methanolysis with 16% HCl in methanol overnight. Following methanolysis, FAMEs were extracted into hexane and isolated by TLC using 80:20 hexane:ether mobile phase. The plate was stained with 2,7-dichlorofluorescein and the FAME band scraped and extracted into hexane. The FAME extract was resuspended in 20 μl of nonane and analyzed by GC-MS.

### FAME analysis by GC-MS

**Fig. 1.**
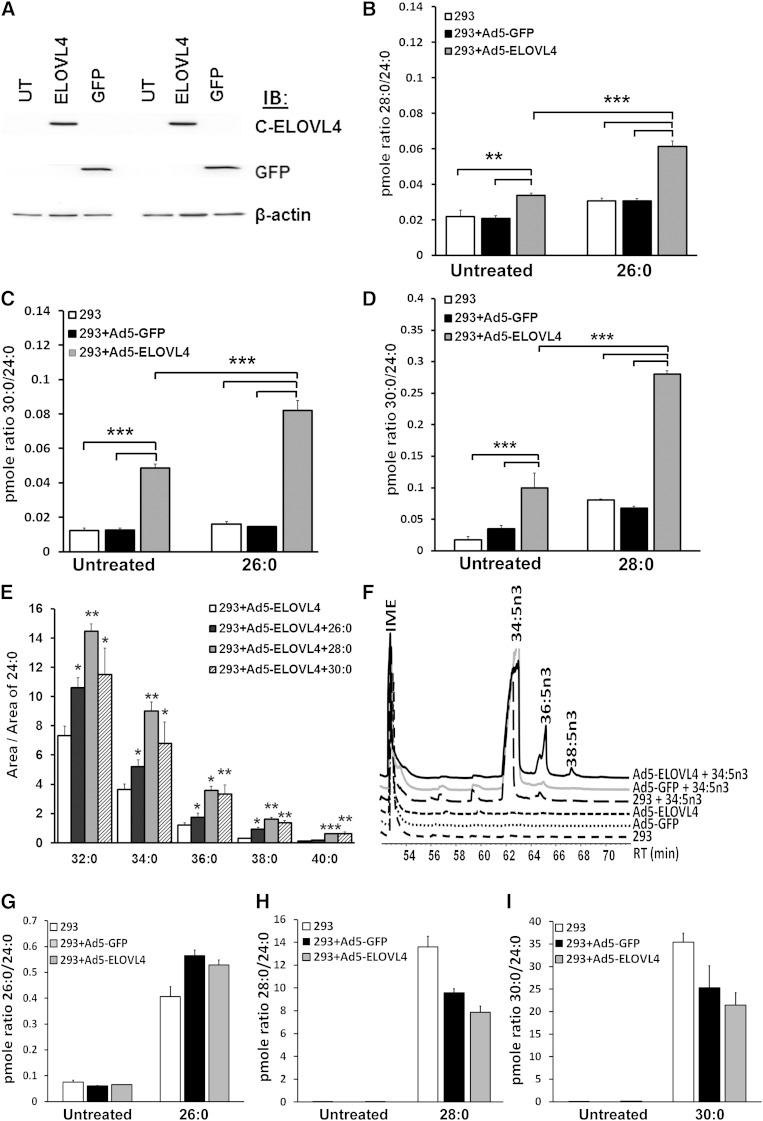
ELOVL4 elongates FAs >C26 to VLC-PUFAs in HEK293T cells. A: Western blotting showing over-expressed ELOVL4 protein detected by C-terminal-specific antibody (C-ELOVL4); GFP and UT controls showed no immunoreactivity to the C-ELOVL4. B: ELOVL4 over-expressing cells supplemented with 26:0 showed significant elongation of the precursor to 28:0 compared with controls. C: ELOVL4 over-expressing cells elongated 26:0 to 30:0. D: ELOVL4 over-expressing cells supplemented with 28:0 significantly elongated the precursor to 30:0 compared with controls. E: VLC-FAs >C32 up to 40:0 were generated in ELOVL4 over-expressing cells supplemented with C26:0, C28:0, and C30:0. Control cells do not have detectable levels of these FAs. Data are represented as ratio of area of FA product generated to the area of 24:0 for normalization across samples. Significance (n = 3) was determined by multivariate ANOVA and post hoc Scheffe test (*P < 0.05; **P < 0.01; ***P < 0.001). F: GC-MS tracing showing elongation of supplemented VLC-PUFA precursor 34:5n3 to 36:5n3 and 38:5n3 in ELOVL4 over-expressing cells. Controls showed no detectable levels of these VLC-PUFA products with or without supplementation. RT, retention time (min). Picomole ratios depicting the levels of internalized substrate 26:0 (G), 28:0 (H), and 30:0 (I) in the treated and untreated groups. IB, immunoblotting.

Picomole values of saturated VLC-FAs (Fig. 1B–D, G–I) were measured via direct comparison to external standards as described in Agbaga et al. (8). VLC-FAs (Fig. 1E) ≥C32 were measured as area counts due to the lack of external standards and were analyzed by GC-MS methodology described in Agbaga et al. (8). VLC-PUFAs (Fig. 1F) represented as GC-MS tracings were normalized to the response of isocholesteryl methyl ether, which was shown to be consistent regardless of experimental differences, and were analyzed by GC-MS methodology described in Yu et al. (35). FA mole percentages (Figs. 4A–D; 5A, B; 6B) were measured directly by GC-flame ionization detector (FID) as described in Yu et al. (35).

### Elongase assay and reverse phase high-performance TLC analysis

Microsomes from HEK293T cells expressing ELOVL4 were prepared and elongase assays performed as per Logan et al. (9). Briefly, elongase activity was assayed in a total volume of 200 μl of TEGM reaction buffer [50 mM Tris (pH 7.5), 1 mM MgCl_2_, 150 μM Triton X-100, 1 mM NADPH, 1 mM NADH, 10 mM β-mercaptoethanol, and 1 mM dithiothreitol], acyl-CoA acceptor (34:5n3-CoA), and 10 μM 2-[^14^C]malonyl-CoA at 37°C for 1 h. Individual reactions were initiated with the addition of 200 μg of microsomal protein. To measure condensing activity alone, NADPH/NADH was omitted from the reaction buffer. FAMEs were spotted on C-18 reverse phase silica high-performance TLC plates (Analtech Inc., Newark, DE) and resolved using CHCl_3_:methanol:water (5:15:1, by vol) as the mobile phase. The plates were exposed to a multi-sensitive storage phosphor screen for 48 h and the radioactivity visualized using a Packard Cyclone Plus phosphor imager (PerkinElmer, Santa Clara, CA). Data were analyzed as per Logan et al. (9).

### Statistical analysis

All experiments were conducted in triplicate independent samples. Statistical differences between experimental groups (n = 3) were analyzed using a multivariate ANOVA followed by a post hoc Scheffe test using Statistica 2000 software (StatSoft Inc., Tulsa, OK). Data are represented as the mean ± SD. Significance is indicated by *P* value measurements with *P* < 0.05 considered significant; *P < 0.05; **P < 0.01; ***P < 0.001.

## RESULTS

### ELOVL4 elongates FAs greater than 26 carbons

ELOVL4 has been shown to elongate 26:0 to 28:0 in culture (8). Longer chain FAs up to 38 carbons were also generated in the cells expressing ELOVL4, but the direct connection between ELOVL4 and these elongation products has not been established. It was hypothesized that ELOVL4 was probably mediating these additional elongation steps. However, the existence of another elongase that could carry the reaction forward from the initial ELOVL4-mediated elongation of 26:0 to 28:0 was not definitively ruled out. We recently showed that ELOVL4 mediated the elongation of 34:5n3 to 36:5n3 PUFA (9); however, catalysis of successive steps from C28 to C34 by ELOVL4 has not been established.

Here we demonstrate that, in addition to elongation of 26:0 to 28:0 (8) and 34:5n3 to 36:5n3 (9), ELOVL4 is involved in the elongation of 28:0 to 30:0 and 30:0 to 32:0. We over-expressed mouse ELOVL4 in HEK293T cells via adenoviral transduction and supplemented with 26:0, 28:0, 30:0, and 34:5n3 (**Fig. 1**). GFP-transduced and untransduced (UT) cells served as controls. Expression of ELOVL4 was analyzed by Western blotting and detected by the previously characterized C-terminal-specific antibody (C-ELOVL4; Fig. 1A) that recognizes the WT ELOVL4 protein (8). When supplemented with 26:0, ELOVL4 over-expressing cells elongated the precursor to 28:0 (Fig. 1B) in concurrence with previously published results (8). Further elongation products of 30:0 (Fig. 1C) up to 40:0 (Fig. 1E) were also generated, but only in ELOVL4-expressing cells. Controls (GFP and UT cells) did not generate any detectable level of these VLC-FAs, C32 to C40. Over-expression of ELOVL4 (gray bars) was sufficient to generate small but significant amounts of the elongation products in the absence of exogenous precursors (“Untreated”), presumably from endogenous long chain precursors (Fig. 1B–D). Controls showed no specific elongation in the presence or absence of the precursor. When supplemented with 28:0 (Fig. 1D, E) or 30:0 (Fig. 1E), ELOVL4 over-expressing cells significantly elongated the precursors to 30:0 (a 3-fold increase from its untreated counterpart) and 32:0 and longer, respectively. There is a low level of endogenous human ELOVL4 mRNA expression in the HEK293T cells, which was detected by quantitative real-time RT-PCR (9). This may account for the low levels of VLC-FA products from the elongation of endogenous substrates. For quantification of VLC-FAs, we determined the ratio of the area of the elongated product to the area of 24:0, shown to be a stable denominator (Fig. 1E). ELOVL4-expressing cells also elongated VLC-PUFA precursors 34:5n3 to 36:5n3 and 38:5n3, while controls showed no detectable levels of these elongated products (Fig. 1F). FA elongation is a multi-step process involving the coordinated activities of elongase and desaturase enzymes. Omega-3 (n3) PUFA substrate such as α-linolenic acid (ALA; 18:3n3) undergoes successive desaturation and elongation to generate 20:4n3 and 22:5n3 by ELOVL5 and ELOVL2 elongases (36, 37). However, the activity of ELOVL4 is dependent on the availability of its immediate substrate C26, which is generated from the elongation of C24 by ELOVL1/3 (25, 37–39). In this study, we have shown that ELOVL4 is involved in successive elongation steps generating VLC-FAs with up to 40 carbon atoms.

### ELOVL4 mutants: expression and subcellular localization

**Fig. 2.**
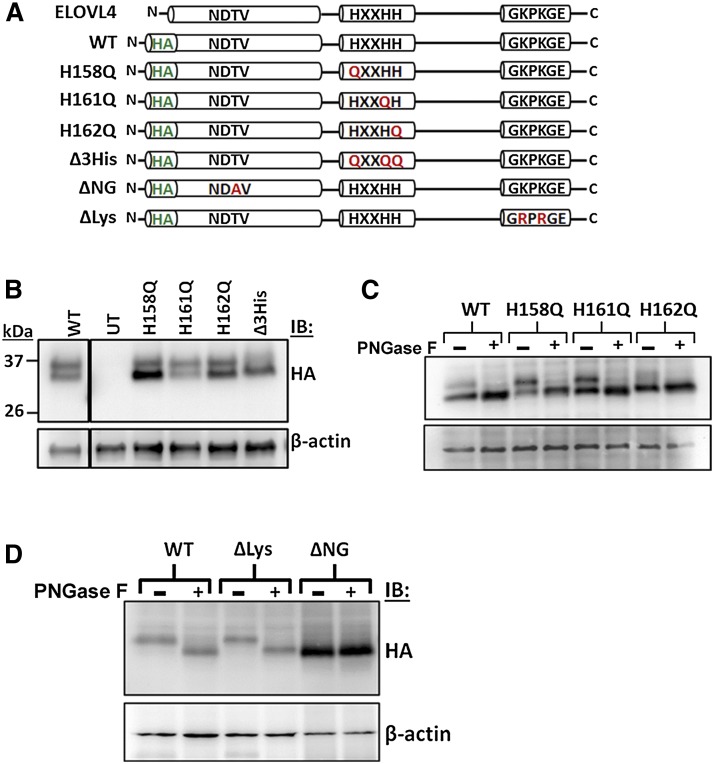
HA-tagged ELOVL4 constructs. A: Schematic representation of untagged (ELOVL4) and HA-ELOVL4 constructs indicating individual mutations in active site (H158Q, H161Q, H162Q, and triple mutant Δ3His), N-glycosylation mutant (T22A; ΔNG), and lysine mutant (K308,310R; ΔLys). B: Adenoviral expression of HA-ELOVL4 constructs in HEK293T cells immunoblotted for HA and β-actin. WT and active site mutants were run on the same gel, albeit separated by several lanes that have been cropped out. C: Cell lysates (20 μg) show a collapse of the double band to a single band in the absence (−) or presence (+) of the N-glycosidase PNGase F indicating N-glycosylation of WT and active site mutants. D: PNGase F-treated lysates show N-glycosylation status of WT and ΔLys, but not ΔNG. IB, immunoblotting.

VLC-FA elongation is affected by mutations in exon 6 of *ELOVL4* (9). However, the dependence of VLC-FA synthesis on the characteristic motifs in ELOVL4 is unresolved. To characterize ELOVL4 activity, mammalian expression vectors carrying HA-tagged mouse *Elovl4* cDNA were constructed (**Fig. 2A**). Four active site mutants were generated by site-directed mutagenesis by substituting histidines with glutamines, H158Q, H161Q, H162Q, and H158,161,162Q (Δ3His). To assess the dependence of ELOVL4 activity on the N-glycosylation status of the protein, we generated an N-glycosylation mutant (ΔNG) by substituting the threonine with alanine (T22A) in the consensus glycosylation site NDTV, similar to the mutation reported by Grayson and Molday (5). To study the effect of ER localization on consequent function, we modified the C-terminal lysines at positions 308 and 310, the positively charged residues that are thought to be recognized by the ER retention machinery, to arginine (K308,310R; ΔLys) to displace ELOVL4 from the ER.

Recombinant adenovirus (*Ad5*) of the HA-tagged constructs was generated by homologous recombination using RAPAd CMV adenoviral expression system (Cell Biolabs, Inc., San Diego, CA). Recombinant adenovirus for untagged ELOVL4 was generated previously as described in Agbaga et al. (8). HEK293T cells were transduced with adenovirus for expression of the constructs (Fig. 2B). Western blotting revealed a double banding pattern of HA-ELOVL4 (WT) at the predicted molecular mass of ∼34–36 kDa. The double bands have been shown to be the result of N-glycosylation of ELOVL4 (9). WT and all active site mutants were glycosylated (Fig. 2C). As expected, ΔNG was devoid of N-glycosylation as evidenced from the resistance to PNGase F digestion, while mutating the C-terminal lysines (ΔLys) did not affect its N-glycosylation status (Fig. 2D).

**Fig. 3.**
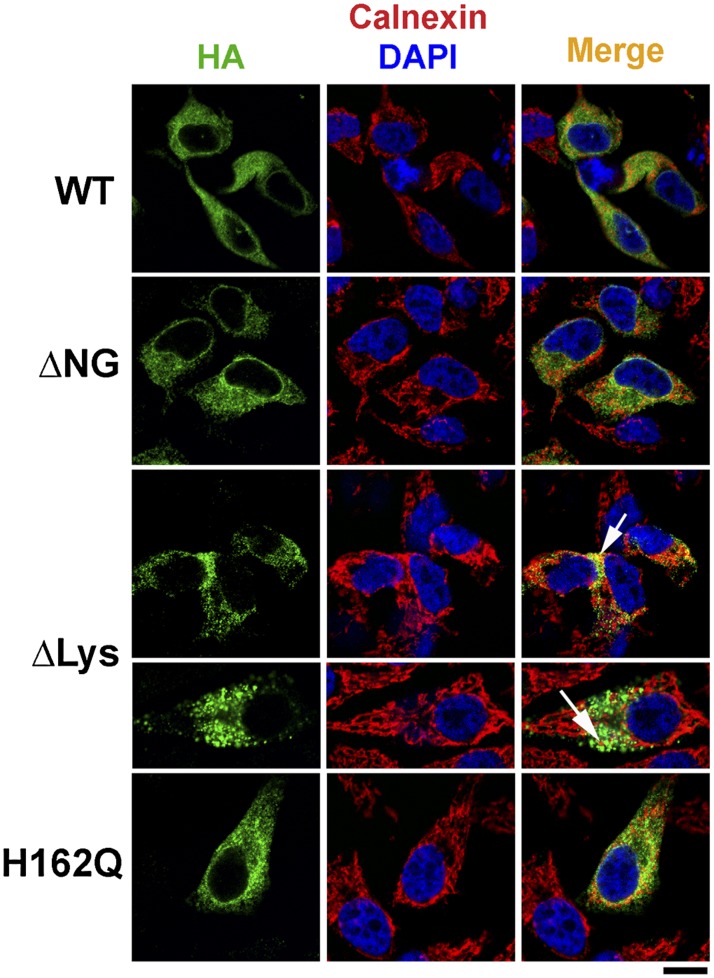
HeLa cells transiently transfected with HA-ELOVL4 constructs show immunoreactivity for HA staining (green) colocalizing with ER marker calnexin (red) in WT, ΔNG, and H162Q expressing cells. ΔLys appears to be more punctate and aggregated (arrows). Scale bar, 15 μm.

To determine the subcellular localization of our ELOVL4 constructs, we expressed them transiently in HeLa cells and examined by immunofluorescence microscopy (**Fig. 3**). HA-immunoreactivity showed colocalization of WT with the ER marker calnexin. ΔNG and H162Q (representative of all active site mutants) showed a similar localization pattern as WT. ΔLys however, showed little colocalization with calnexin while mostly displaying aggregated and punctate staining (arrows). Because these are static images, it is conceivable that there may be some amount of the ΔLys protein being synthesized and transiting the ER, thereby displaying the colocalization with calnexin.

### ELOVL4 activity is dependent on active site histidines

**Fig. 4.**
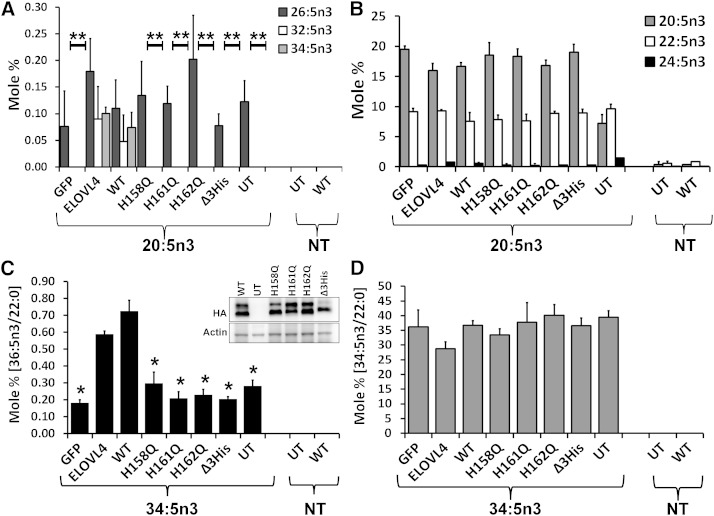
ELOVL4 active site mutants are deficient in VLC-PUFA biosynthesis. A: Elongation of 20:5n3 in HEK293T cells to 32:5n3 and 34:5n3 in ELOVL4 and WT, but not in catalytic dead mutants (histidine mutants) or GFP-expressing and UT controls. B: Relative mole percent of 20:5n3, 22:5n3, and 24:5n3 with and without (NT) supplementation showing comparable levels of these FAs across samples in transduced HEK293T cells. C: Elongation of 34:5n3 to 36:5n3 normalized to 22:0 in HEK293T cells expressing ELOVL4 and WT, but not in active site mutants, which were comparable to controls (GFP and UT). Data are represented as the mean ± SD (n = 3). Significance was assessed in comparison to WT; *P < 0.05; **P < 0.01. (Inset: adenoviral-mediated expression of HA-ELOVL4 proteins supplemented with either 20:5n3 or 34:5n3). D: Levels of 34:5n3 internalized across treated and NT samples showing comparable levels of the precursor.

**Fig. 5.**
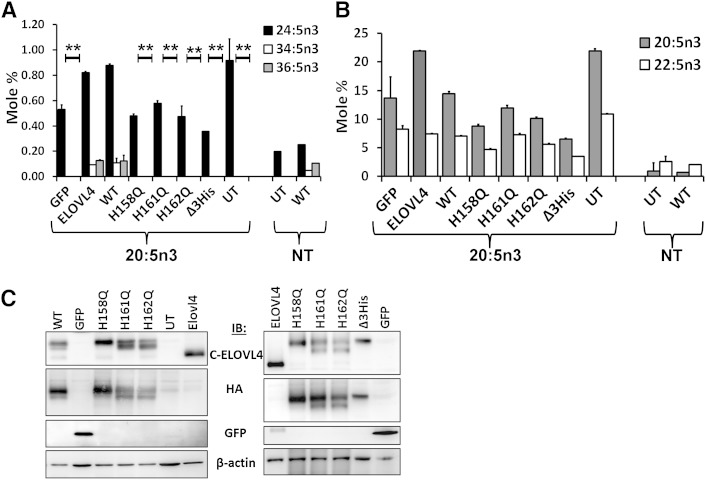
ELOVL4 active site mutants lack elongase activity in Arpe19 cells. A: Elongation of 20:5n3 to 34:5n3 and 36:5n3 in ARPE19 cells expressing ELOVL4 and WT, but not in active site mutants or controls. Data are represented as the mean ± SD (n = 3). Significance was assessed in comparison to WT activity (**P < 0.01). B: Relative mole percent of 20:5n3 and its immediate elongated products 22:5n3 and 24:5n3 in ARPE19 cells with and without supplementation. C: Representative Western blots of ARPE19 cells showing comparable levels of untagged (detected by C-ELOVL4 antibody) and HA-tagged (detected by both C-ELOVL4 and HA) ELOVL4 protein expression. Blots were reprobed for GFP and normalized to β-actin. IB, immunoblotting.

To establish ELOVL4 as the elongase in VLC-PUFA elongation, we generated catalytic dead mutants by substituting the iron-coordinating histidine residues in the putative active site with glutamine. We over-expressed ELOVL4, HA-ELOVL4 (WT), and active site mutants (H158Q, H161Q, H162Q, and Δ3His) in HEK293T cells and supplemented with or without [nontreated (NT)] 20:5n3 (**Fig. 4A, B**), previously shown to be the preferred substrate for VLC-PUFA synthesis (35). UT and GFP-transduced cells served as controls. Only ELOVL4 and WT were able to elongate 20:5n3 to n3 VLC-PUFA, with 34:5n3 being the predominant elongated product (Fig. 4A). Active site histidine mutants could not elongate the FA precursor and were comparable to controls. FA elongation products that were not dependent on ELOVL4 (<C28), including the immediate VLC-PUFA precursor C26, were comparable in all experimental groups (Fig. 4B). ELOVL4 and WT elongated the precursors to a similar extent, confirming that the tag does not interfere with ELOVL4-mediated VLC-PUFA synthesis. Supplementation with 34:5n3 resulted in significant elongation of the precursor to 36:5n3 in HEK293T cells over-expressing ELOVL4 and WT (Fig. 4C). Active site mutants were not able to elongate the precursor beyond background levels and were comparable to controls (GFP and UT). Levels of the precursor 34:5n3 were comparable across samples (Fig. 4D). These data are represented as mole percent normalized to an endogenous FA (22:0), whose levels were unaffected by ELOVL4 over-expression. Elongation of VLC-PUFA by ELOVL4 was recapitulated in another cell line, retinal pigment epithelium cells (ARPE19) supplemented with 20:5n3 (**Fig. 5A–C**). Irrespective of the difference in the FA profile of ARPE19 cells from HEK293T cells, they were able to elongate the precursor (20:5n3) to VLC-FAs (34:5n3 and 36:5n3) upon over-expression of ELOVL4 (Fig. 5A). Active site mutants were deficient in these VLC products, albeit they were able to generate comparable levels of the immediate elongation products (22:5n3 and 24:5n3) of the precursor (Fig. 5B). Irrespective of the comparable expression levels of the active site mutants compared with WT (Fig. 5C), the active site mutants were devoid of any detectable activity in generating the VLC-FAs.

These experiments demonstrate conclusively that the enzymatic activity of ELOVL4 is mediated by the histidine residues in the active site.

### ELOVL4 activity requires retention to the ER, while N-glycosylation is dispensable

**Fig. 6.**
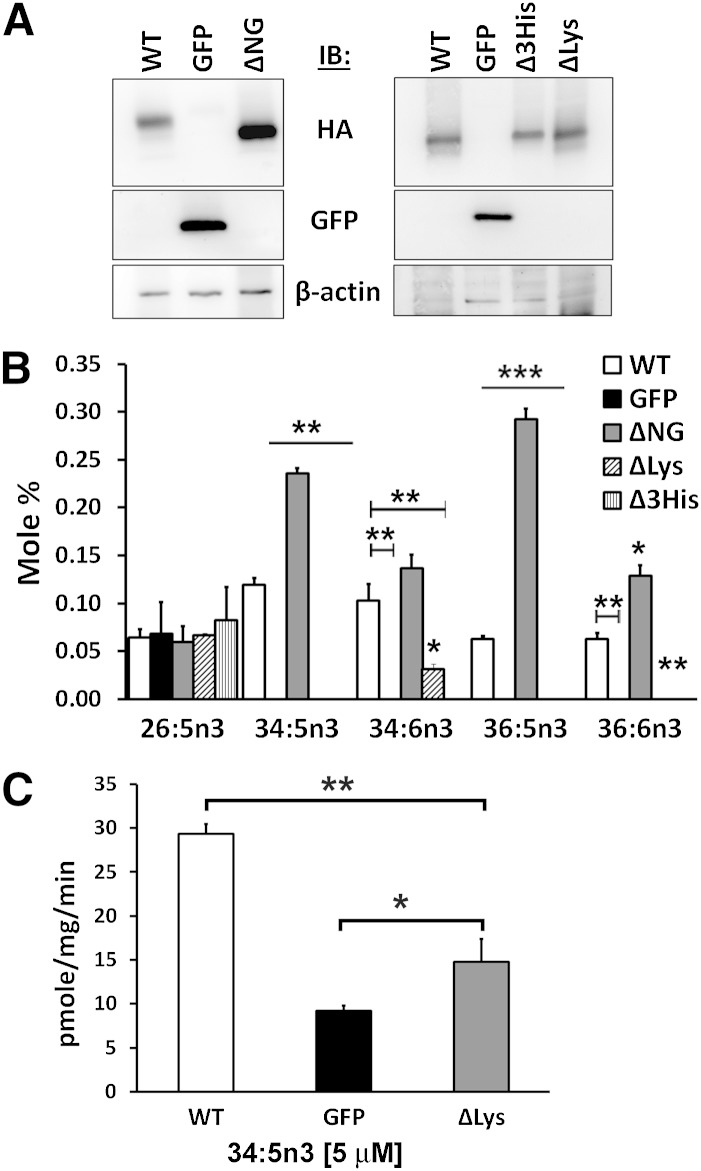
ELOVL4-mediated biosynthesis of VLC-PUFAs is independent of N-glycosylation but requires retention in the ER. A: Western blot showing increased expression of ΔNG compared with WT (left panel) and comparable levels of Δ3His and ΔLys to WT (right panel) in ARPE19 cells. Blots were reprobed for GFP and β-actin. B: ΔNG showed increased levels of 34:5n3 and 36:5n3 elongation products compared with WT in ARPE19 cells supplemented with 20:5n3, correlating with increased expression levels of the protein. ΔLys and Δ3His did not show any detectable level of the elongated VLC-PUFA products. Data are represented as the mean ± SD (n = 3); significance was determined in comparison to WT; *P < 0.05; **P < 0.01; ***P < 0.001. C: Quantification of the condensation activity in the microsome elongase assay of WT, GFP, and ΔLys samples in the presence of 5 μM of 34:5n3-CoA shows a significant reduction in the generation of the 3-keto-acyl intermediate in the ΔLys microsomes. However, there was some residual activity above background (GFP). Data are represented as the mean ± SD (n = 3). Significance was assessed using Student's *t*-test (*P < 0.05; **P < 0.01). IB, immunoblotting.

ELOVL4 is N-glycosylated, a posttranslational modification known to be important for the proper folding and stability of proteins, albeit the relevance of this glycosylation for enzyme activity is unclear. We over-expressed ΔNG mutant and WT in ARPE19 cells and supplemented with 20:5n3 (**Fig. 6A, B**). Western blotting showed twice as much over-expression of the ΔNG mutant compared with WT (Fig. 6A; left panel). Both WT and ΔNG mutants were able to elongate the precursor to VLC-PUFA (Fig. 6B). GFP-expressing control cells showed no specific elongation. The mole percent of 34:5n3 VLC-PUFA elongation product in ΔNG mutant was observed to be twice as much as WT, correlating with the higher level of expression of the ΔNG protein. Levels of C26, not affected by ELOVL4 expression, were comparable in all experimental groups. Thus, the lack of N-glycosylation did not adversely affect ELOVL4 enzyme activity.

Mutations in ELOVL4 result in truncation and loss of the C terminus of the protein, the region encoding the dilysine ER-retention signal, and subsequent loss in enzyme activity (1–3, 9). Whether the lack of activity of the truncated protein is due to its mislocalization or to some structural modifications resulting from the mutation is unclear. Thus, to determine if localization of ELOVL4 to ER membranes is critical for protein function, we over-expressed the ΔLys mutant and WT in ARPE19 cells and supplemented with 20:5n3 (Fig. 6A, B). Western blotting revealed comparable levels of expression of the ΔLys mutant compared with WT (Fig. 6A; right panel). GFP and catalytic dead mutant Δ3His-expressing cells served as controls. WT elongated the precursor to VLC-PUFAs, with 34:5n3 being the predominant product followed by 36:5n3. These products were further desaturated to yield 34:6n3 and 36:6n3, respectively. In contrast, ΔLys mutant did not synthesize the aforementioned VLC-PUFA products (Fig. 6B). There was a marginal amount of the desaturated product 34:6n3 detected in ΔLys samples, which could have been generated from 34:5n3 (undetected) synthesized by the ΔLys mutant as it was transiting through the ER.

During FA elongation, ELOVL4 mediates the first rate-limiting condensation reaction between malonyl-CoA and a fatty acyl-CoA to generate the 3-keto-acyl intermediate (9). Other enzymes in the ER carry the reaction forward through subsequent reductions and dehydration to generate the VLC-FA elongated by two carbons. We measured the condensation activity of the ΔLys mutant using the in vitro cell-free microsome assay as described in Logan et al. (9), where we determined that the condensation activity of ELOVL4 was optimal at 5 μM of 34:5n3-CoA substrate. We compared the ability of WT ELOVL4 and ΔLys with an intact catalytic core to mediate the condensation of 34:5n3-CoA. Mislocalization of the full-length protein by mutating the C-terminal lysine residues significantly decreased its condensation activity, albeit some activity above background still remained (Fig. 6C). This could be the result of residual ΔLys protein transiting the ER as evidenced earlier in the cell-based assay. The decrease in the generation of the 3-keto intermediate by the ΔLys mutant suggests that mislocalization of ELOVL4 to compartments other than the ER is sufficient for loss of enzyme activity. We show that the C-terminal lysines are required for retention of ELOVL4 in ER membranes and are thus necessary for the elongation of VLC-FAs.

## DISCUSSION

Human patients with mutations in *ELOVL4* develop Stargardt macular dystrophy (STGD3) characterized by early onset retinal degeneration and loss of vision (1–3). The dominant nature of the disease suggests a role of the mutant protein in disease manifestation, but does not preclude the importance of the VLC-PUFA products catalyzed by ELOVL4 in the retina. To determine the role of the truncated protein in STGD3 pathogenesis, we need to have a better understanding of WT ELOVL4 activity. We therefore focused our attention on developing a comprehensive characterization of WT ELOVL4 to provide a solid platform to compare mutant activity and function in VLC-PUFA synthesis.

Although ELOVL4 was shown to elongate 26:0 to 28:0 (8), whether it mediated subsequent elongation steps generating VLC-FA species ranging up to 40 carbons in length was unclear. Mouse models of ELOVL4 (heterozygote knock-in and KO) show reduced levels of VLC-PUFA in the retina, while homozygous mice die shortly after birth from dehydration due to compromised skin barrier permeability from lack of ω-O-acylceramides containing VLC-FAs (21). These studies, however, do not unequivocally refute the existence of another elongase whose activity may be dependent on ELOVL4-mediated generation of its precursor. In this study, we demonstrated that ELOVL4 is indeed capable of successive elongation steps mediating the rate-limiting condensation reaction. Denic and Weissman (40) demonstrated a molecular caliper-like mechanism whereby the FA chain lengths catalyzed by yeast elongase, Elop, are determined by the distance between the active site and the juxta-luminal lysine residue. Whether this mechanism is applicable to ELOVL4 and the possibility of skewing synthesis to a preferred carbon chain length by ELOVL4 remains to be tested. While we addressed, for the most part, ELOVL4-mediated elongation steps, the limitations in our assays were reflective of the lack of commercially available VLC-FA and -PUFA substrates. Thus, we propose that ELOVL4 is most likely also involved in the elongation of C32 to C34 FAs, but were unable to test this due to the lack of available substrate.

Mutagenesis experiments with mammalian stearoyl-CoA desaturase have established the role of iron-binding conserved histidines in the catalytic core as essential for enzyme activity, while substitution of nonconserved flanking histidines had no effect on enzymatic activity (41). These results were later recapitulated in similar experiments on cyanobacterial DesA desaturase (42) and on *Pseudomonas* monooxygenases AlkB (43) and XylM (44). In agreement with these studies, substitution of any of the histidine residues in the catalytic core of ELOVL4 resulted in a complete loss of enzymatic activity. Lack of activity in the active site mutants argues against the hypothesis that ELOVL4 may be a cofactor. Protein cofactors typically exert their effects through protein-protein interactions involving multiple residues. Based on these reports and taken together with localization and stability of expression of the ELOVL4 active site mutants comparable to WT ELOVL4, we do not suspect global misfolding of the proteins to be the cause of lack of enzymatic activity. However, we cannot rule out the possibility of localized alterations in structure in the vicinity of the amino acid substitutions in the active site mutants.

Elongation of VLC-FAs is a process that occurs in the ER, which is the site for posttranslational modifications such as N-glycosylation that is widely accepted to be required for structural stability and protein folding. Mutations affecting the N-glycosylation status of rhodopsin result in retinitis pigmentosa (RP). Rhodopsin is dually glycosylated at N2 and N15 (45), and mutations in these residues or the neighboring consensus residues (T4K, N15S, and T17M) have been linked to a subset of RP called sector RP, primarily affecting the inferior retina (46–49). Over-expression of the human N-glycosylation mutations in *Xenopus laevis* revealed that, while N-glycosylation was not crucial for rhodopsin biosynthesis or trafficking, it did affect photoreceptor viability (50). However, similar bovine rhodopsin mutations did not affect rod photoreceptor viability (50). Thus, species-specific attributes of N-glycosylation govern its effect on photoreceptor survival and function. Over-expression of the mouse ELOVL4 deficient in N-glycosylation in our cell-based system did not overtly affect localization or function. Synthesis of VLC-PUFA by ELOVL4 deficient in N-glycosylation (ΔNG) suggests that the overall three-dimensional structure of the protein is intact and that N-glycosylation, at least for mouse ELOVL4, is dispensable. It also suggests that interactions between ELOVL4 and other members of the FA elongation complex are independent of the N-glycosylation status of the protein. Whether there are species-specific effects of N-glycosylated ELOVL4, its effect on photoreceptor structure, viability, and function remain to be explored. It is conceivable that there may be photoreceptor-specific cellular factors, absent in our cell-based system, which regulate the glycosylation status of ELOVL4 and alter its activity and VLC-PUFA synthesis. Further experimentation with glycosylation-deficient ELOVL4 in an animal model would shed light on the importance of this posttranslational modification on VLC-PUFA synthesis.

STGD3 mutant protein has been shown to be mislocalized in cultured cells (6, 7, 51), as well as in transgenic pig retina (52). To understand whether mislocalization has any effect on ELOVL4 function, we generated the ΔLys mutant. Lysine residues juxtaposed to the C terminus of proteins have been shown to be necessary for retention of transmembrane proteins in the ER (33) and are thought to be recognized by the ER retrieval machinery. By mutating these C-terminal lysines, we have shown mislocalization of WT ELOVL4. Another intriguing finding was the N-glycosylation of the ΔLys mutant, which suggests that this posttranslational modification might be an early event prior to the mislocalization from the ER. In our cell-based assay, we observed a loss of VLC-PUFA synthesis when supplemented with 20:5n3 in culture in the ΔLys mutant compared with its WT counterpart. Thus, retention to ER membranes is necessary for the production of VLC-PUFAs by ELOVL4, despite the fact that the ΔLys mutant has an intact catalytic core. This suggests that when ELOVL4 is mislocalized to other membranous compartments: *1*) access to the precursor is lost; *2*) association with the other components of the elongation machinery, for example KAR and TER, might be disbanded; or *3*) both. These findings suggest that the mere mislocalization of the STGD3 mutant would render it inactive. However, we have shown that even when the STGD3 mutant was forced to localize to the ER, it lacked condensation activity (9). This suggests that structural changes due to the mutation and subsequent modified amino acids are responsible for the lack of activity in the STGD3 mutant.

In conclusion, we have demonstrated the prominent features of ELOVL4 for optimal enzyme activity during biosynthesis of VLC-FAs. More importantly, our analysis of ELOVL4 enzyme activity paves an avenue to study this unique class of FAs, the VLC-PUFAs, which have long been overlooked and undervalued.
